# The Biology of the Genus *Ceiba*, a Potential Source for Sustainable Production of Natural Fiber

**DOI:** 10.3390/plants11040521

**Published:** 2022-02-15

**Authors:** Ximena Gómez-Maqueo, Alicia Gamboa-deBuen

**Affiliations:** Instituto de Ecología, Universidad Nacional Autónoma de México, Mexico City 04510, Mexico

**Keywords:** kapok, *Ceiba*, fiber, sustainability, Malvaceae, silk-cotton

## Abstract

The species of the genus *Ceiba* produces fruits with fibers with a high content of cellulose. The fiber is used for textiles, cushion filling and for industrial purposes and its characteristics have been studied in some species including *Ceiba pentandra* (kapok), *C. speciosa* and *C. aesculifolia*. The use of the trunk and seeds of *Ceiba* has also been described for different species. This article presents a review on the biological diversity of the genus Ceiba (Malvaceae). The genus *Ceiba* has 18 recognized species that are distributed naturally in America and Africa. However, some *Ceiba* trees have been introduced to various countries, especially in Asia, due to their ornamental interest and potential uses for their fiber. Ecophysiological studies of different *Ceiba* species have shown that resistance to adverse environmental conditions varies from species to species. Therefore, *Ceiba* species are considered potentially useful in restoring ecosystems impacted by human activity. The information related to the classification, morphological characteristics, phenology, ecophysiology and distribution of the different species will be extremely relevant for the sustainable production of kapok fiber. Finally, the recent genomic and transcriptomic studies also provide a valuable resource for further genetic improvement and effective use of *Ceiba* trees.

## 1. Introduction

Natural fibers are obtained from different plants and animals and have many uses, both locally and industrially. Although the popularization of oil-based fibers has reduced the historic demand of natural fibers, with synthetic fibers holding about 58% of total fiber use by 2013, it is estimated that production of natural fibers worldwide corresponded to approximately 33 million tons by the same year. About 96% of all natural fibers produced are derived from plants, with cotton accounting for 79% of total production, while other plant-based fibers, such as jute, hemp, sisal, coir or kapok, contribute to about 18% [1].

In recent years, there has been increasing concern on the sustainability and negative impacts on the environment posed not only by oil-based fibers, but also by fibers naturally sourced or regenerated from cellulose [2]. For instance, cotton is the most widespread plant-based fiber; about 80 countries produce cotton commercially, occupying approximately 2.5% of the farmable land around the world [1]. Still, cotton production requires about 25% of the total insecticide and 10% of total pesticides produced worldwide, as well as intensive irrigation with an estimated 7000–29,000 L of water required to produce 1 kg of cotton [3]. Meanwhile, the other fiber-producing species are exploited at smaller scales around the globe and could open an opportunity to cope with the need of sustainable and environmentally friendly production of natural fibers. Some of these alternative fibers, such as kapok, need to be mixed with other fibers to be properly spun for textile production. However, by incorporating kapok in the production of other synthetic or cotton fibers, it could be possible to reduce the total environmental footprint of the finished products.

**Figure 1:**
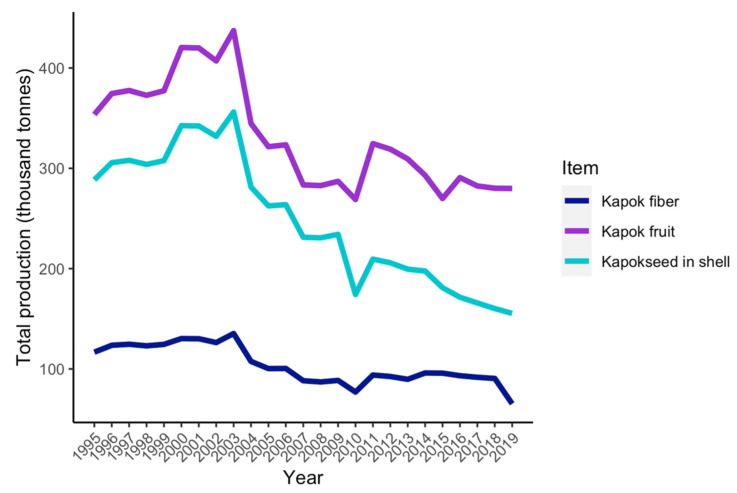
FAO estimates of worldwide production of kapok fiber, fruits and seeds since 1995. The data correspond to the combined production of Indonesia and Thailand, the only countries indicated in the database.

“Kapok” is a common term used to identify a type of “seed fiber” (e.g., cotton) produced mainly by two plant species known as “kapok trees”: *Bombax ceiba* L. (Malvaceae Juss., also known as “red cotton tree” or “red silk-cotton”), and *Ceiba pentandra* (L.) Gaertn. (Malvaceae, also known as “silk-cotton” or “Java cotton”). There is usually confusion in the literature as to which of the two species is used as the source of “kapok”, though most of the information regarding the physical and chemical properties of kapok fibers comes from *C. pentandra*. It is noteworthy to mention that cotton, jute and kapok, three of the main fiber-producing plants, belong to the Malvaceae family. As observed in taxonomic revisions of the subfamilies within the Malvaceae, there are many other genera aside from *Bombax* L. and *Ceiba* Mill. that belong to the “kapok clade” and produce floss-bearing fruits, such as the genera *Pachira* Aubl., *Eriotheca* Schott and Endl. or *Pseudobombax* Dugand [4], though the viability of producing fiber or cellulose from these species remains to be explored. *C. pentandra* is cultivated and found widely spread in several plantations in Southeast Asia, where most of the countries that produce, and export kapok fibers are located. Before WWII, kapok trees were an important cash crop because the fiber was extensively used in life jackets and aviation clothing, among other things. In the 1960s, as a result of the massive production of synthetic fibers, the kapok trade declined substantially [5]. By 2015, Indonesia was one of the largest producers and exporters of kapok in the world, followed by Thailand, which are the two countries on the FAO database [6,7]. Over the past 10 years, there has been an increasing interest in studying the properties of kapok. This fiber has been found to be environmentally friendly and biodegradable as well as having anti-bacterial and anti-mite properties. Most of the research that has been carried out is related to materials science and engineering. For example, potential applications of kapok fiber are related to oil sorbents [6]. Still, the production of kapok fiber has had a slight declining trend, as seen in Figure 1 [7].

Aside from the economic importance of *C. pentandra*, several species from the genus are regarded as culturally and environmentally important species; they are found incorporated in local rituals almost everywhere [8]. In Mexico, the Mayan and Aztec pre-Hispanic cultures considered *C. pentandra* and *C. aesculifolia* (Kunth) Britten and Baker f. respectively, as sacred trees that connected the human world with the place where the Gods lived [9,10,11]. In Guatemala, *C. pentandra* is considered the national tree [12]. In west Africa, *C. pentandra* is also regarded as a sacred tree [8], while in Asia, cultivated kapok offers an important source of seasonal jobs and income for the kapok-producing countries [1]. In this review we will focus on *C. pentandra* and other species of the genus *Ceiba* as potential sources of sustainable kapok, as well as other ecological and cultural benefits.

## 2. Characteristics of the Genus *Ceiba*

**Figure 2:**
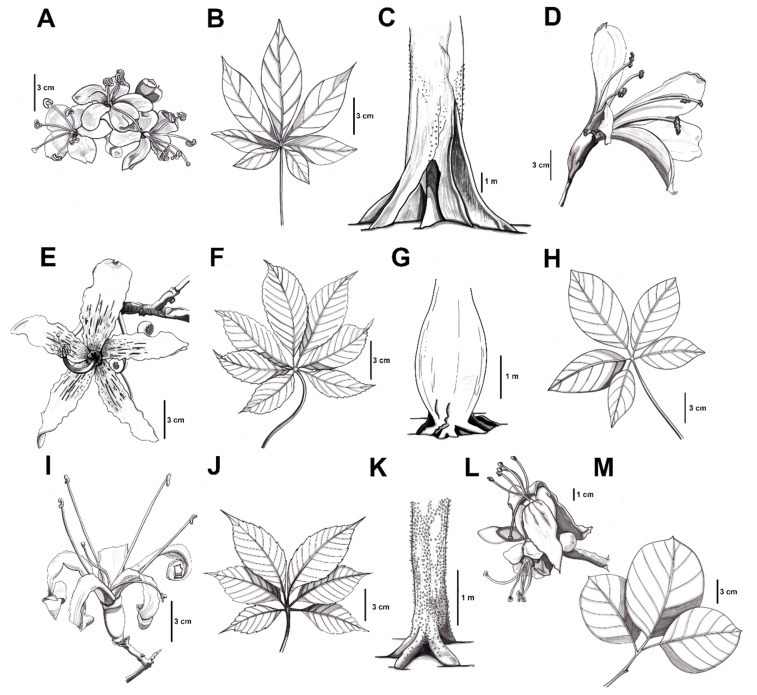
Representative illustrations of the diversity of flowers, leaves and trunks seen within the genus *Ceiba*. (**A**–**C**): *C. pentandra*; (**D**,**H**): *C. samauma*; (**E**–**G**): *C. speciosa*; (**I**–**K**): *C. aesculifolia*; (**L**,**M**): *C. jasminodora*.

The genus *Ceiba* comprises 18 species, 17 of which are naturally distributed in the Neotropics. *Ceiba* species are trees usually between 10 to 25 m tall, with some species reaching above 50 m (*C. pentandra*, *C. lupuna* P.E. Gibbs and Semir), or as small treelets of about 2 m tall, such as like *C. jasminodora* (A. St.-Hil.) K. Schum. [13]. They present digitate composite leaves with serrated or plain borders, and characteristic aculeate trunks and branches [14]. Some species, such as *C. chodatii* (Hassl.) Ravenna, *C. speciosa* (A. St.-Hil.) Ravenna, *C. glaziovii* (Kuntze) K. Schum., and *C. pubiflora* (A. St.-Hil.) K. Schum., present ventricose or “swollen” trunks, which explain some of the common names given to these trees in South America, such as “palo borracho” due to the bottle-shaped trunk or “barriguda” as in having a swollen belly [14]. The flowers present a corolla conformed by five petals, which are diverse in size and color, ranging from pale tones, such as white, ivory, yellow or light pink, to vibrant colors, such as pink or red, and sometimes with yellow tones towards the base. In some cases, the petals present dark colored striations. The fruits are woody capsules that contain a modified endocarp into long, tubular trichomes that constitutes the kapok fiber, in which the seeds are imbedded. This fiber facilitates seed dispersion through wind. Due to the diversity of flower morphology, *Ceiba* species are pollinated by several species of bats, butterflies, bees and hummingbirds [14]. In Figure 2, we present some representative illustrations of the different flowers, leaves and trunk shapes found within the genus. Most species are deciduous and flower when leafless; the flowers are usually short-lived and present crepuscular anthesis. These characteristics have hampered the taxonomic efforts of identification and classification of the clade [14]. In Table 1, we present a short compilation of morphological characteristics and habitat distribution for each species.

## 3. Ecology of *Ceiba* Species

### 3.1. The Ecosystem and the Challenges Faced by Ceiba Species Due to Habitat Loss and Degradation

The seasonally dry tropical forest (SDTF), in which most *Ceiba* species are present (Table 1, 13 species), is characterized by several months of severe drought (with rainfall less than 100 mm) [18,19]. These types of tropical forests usually have a mean annual temperature above 17 °C and annual rainfall ranging from 250 to 2000 mm, which occurs mostly during six to eight months [19]. Unlike savannahs, which generate under the same climatic conditions and are dominated by a xeromorphic, fire-tolerant grass layer, the SDTF are tree-dominated and have an almost continuous canopy [18]. These ecosystems occur in disjunct floristic nuclei that show high levels of beta diversity [20]. Although covering about 42% of the tropical ecosystems worldwide [19], it is currently one of the most endangered ecosystems by deforestation in order to allocate arable land due to the fertility of the soil, as well as other land uses [18]. Some species, such as *C. rubriflora* and *C. jasminodora*, are also endangered due to habitat loss; the former been endemic to calcareous outcrops in the Serra do Ramalho (Brazil) [15], and the latter being restricted to rocky outcrops in the Espinhaço mountain range (Brazil) [13]. 

The water-availability cycles experienced by plants in the SDTF drives fundamental phenological transitions that limits growth and reproduction to the wet season [19,21]. Additionally, seed germination, seedling establishment and regeneration respond to these water cycles [22]. Most of the species that inhabit in the SDTF shed their leaves during the dry months in order to deal with water limitations [22] and abiotic factors, including certain levels of drought or increases in temperature, which may trigger important transitions such as flowering [21]. Thus, disruption or alteration of these factors by human activity and habitat loss may bring about shifts in phenological transitions, disrupting pollination and reproduction, an event already documented in *C. aesculifolia* [23]. Temperature increases have caused altitudinal shifts in seedling establishment and survival in *C. aesculifolia*, especially in zones near urban settlements in Morelia, Michoacán (Mexico) [24].

According to Pezzini et al. [13], four *Ceiba* species (*C. pentandra*, *C. crispiflora*, *C. samauma* and *C. speciosa*) can also inhabit humid tropical forests, and are associated with river valleys and flood zones or grow within gallery forests. Other sources indicate that *C. schottii* can be found in semi-evergreen forests and mangroves within the Yucatán peninsula in Mexico [17]. Finally, *C. lupuna* is the one species restricted to humid tropical forests [13]. In the Peruvian and Brazilian Amazon, wild populations of *C. pentandra* are threatened by intensive exploitation by the plywood industry [25]. Currently, *C. soluta* and *C. crispiflora* are present in the IUCN red list of threatened species [26]. As in the case of SDTF, humid tropical forests face important challenges associated with climate change that impact on fundamental ecosystem properties, including nutrient cycling, carbon storage, shifts in temperature and rain regimes, that will ultimately have negative impacts on diversity and ecosystem services [27].

### 3.2. Conservation and Ecosystem Restoration Potential of Ceiba Species 

Several *Ceiba* species have been shown to play an important role within their respective ecosystems, which in turn make them suitable for conservation and restoration programs. In this section we will present *C. aesculifolia* and *C. pentandra* as case studies, since most *Ceiba* species are largely understudied.

#### 3.2.1. *Ceiba aesculifolia*

The research performed in several populations of *C. aesculifolia* throughout the Mexican SDFTs recognizes the species as a pioneer-secondary species, which can grow in shallow soils and higher hydric stress [28,29], as well as in low-soil P concentrations [30]. The seedlings have been shown to resist drought through high sapwood water storage capacity, and although this trait renders them vulnerable to xylem embolism, the buffering role of water storage allows the seedlings to maintain their water potential above the soil’s potential as water stress intensifies [31]. Adult trees of this species seem to be resistant to anthropogenic disturbances related to gap formation, such as removal of branches or neighboring vegetation, since there are no differences in growth or density in contrast to individuals in undisturbed areas [32]. The seedlings have better survival to high temperatures in contrast to other local species [24,33], attributes which might play an important role in the implementation of strategies towards conservation, considering the challenges posed by climate change. Still, the concerns raised by [24] Valle-Díaz et al. (2009) regarding the need to assist plant regeneration in disturbed areas near the city of Morelia (Mexico) implies the need of in situ and ex situ plant propagation. To this end, several studies have been conducted on seed germination in wild populations found in Veracruz (Mexico). Recent field work and greenhouse experiments by Velázquez-Rosas et al. [28], Martínez-González et al. [29] and Martínez-González et al. [34] were centered on the importance of seed size variation during germination, during seedling establishment and seedling survival to foliar damage, respectively. Seed size seems to have an effect on germination of individuals emerging in pastures (disturbed areas), whereas there was no effect of seed size in conserved SDTF patches [29] or in greenhouse germination tests [28]. Seedling survival after six months post-germination did not show an association to seed size either [29]. Still, greenhouse experiments showed that seed size does have an effect on total dry weight increase of the seedlings by means of shifts in the root–shoot ratios and changes in leaf area. Total chlorophyll seems to also respond to both seed size and foliar damage [34]. Furthermore, a study performed by Olvera-Mendoza et al. [35] evaluated the genetic diversity within the introduced individuals in the restoration effort conducted by Valle-Díaz et al. [24]. The genetic diversity estimated in the introduced individuals was higher than either of the provenances from which seeds were sourced. This higher diversity could offer the opportunity for reintroduced populations to adapt to an ever-changing environment. Moreover, germination tests performed under field and controlled conditions have demonstrated the positive effects of natural and matrix priming treatments on *C. aesculifolia* seeds (Gómez-Maqueo et al. [36], and references therein). This germination and seedling survival research will help to develop better restoration and conservation strategies of this species as well as provide insight into a sustainable exploitation of this tree by in/ex situ propagation. 

#### 3.2.2. *Ceiba pentandra*

Conservation and restoration efforts in Africa and Asia propose the species as a suitable tree despite being an introduced species. In Madagascar, one proposal stems from field observations of several vertebrate species that feed from or within the trees. These vertebrates eat the flowers, or the insects found in the trees, while some others use the tree as refuge. Several of these vertebrates are effective seed dispersers of the native flora, thus the strategic planting of *C. pentandra* trees could aid in seed dispersion by allowing the movement of animal dispersers over the tree canopy and between forest fragments [37]. Meanwhile in India, research has been conducted on the tolerance to salt stress during germination [38]. The authors indicate that *C. pentandra* is moderately tolerant to salt stress during germination, with mild effects on development and growth. However, soil salinity decreased final germination as well as root and shoot development when the electrical conductivity of the soil exceeded 9 dSm^−1^, with severe effects experienced from 12 to 15 dSm^−1^. Moreover, in India, *C. pentandra* is suitable for sustainable management of agro-forest systems and afforestation field experiments aiming to reclaim degraded coastal farmlands and to increase the productivity of these degraded soils. This field work has offered insight into viable strategies for management of these degraded soils, while also using a multi-purpose species that offers an important source of pollen for local beekeepers, as well as fibers, oils and cattle feed that sustain local livelihood [39]. 

## 4. A Brief History on the Origin of the Clade *Ceiba* and the Arrival of Cultivated *C. pentandra* to Asia

Nowadays, several *Ceiba* species have been introduced worldwide (Figure 2). In east Africa and Asia, the main purpose was for exploitation of different products derived from these plants [5,8], while in the northern hemisphere, they are mostly regarded as ornamental plants in gardens and botanical collections [40,41]. There has been historical uncertainty on whether the genus *Ceiba* originated in the Neotropics or in west Africa [8,42], and even some claims of an Asian origin due to its long history of exploitation [43,44]. As presented in Figure 2, all recognized species are present in America, supporting a Neotropical origin [14,15]. The uncertainty arises from the fact that *C. pentandra* is naturally distributed in both America and Africa, with fossil records of pollen grains about 13,000 years old present in Ghana, suggesting that the species was present prior to any evidence of human-facilitated dispersion in Africa [42]. Although the usual explanation for disjunct distribution of flora between South America and Africa involves an origin of the clade prior to the separation of Gondwana about 96 million years ago (Ma), the study by Dick et al. [42] tested several vicariance hypotheses to explain the disjunct distribution of *C. pentandra*, finding evidence for one of the few cases of extreme long-distance dispersion from the Neotropics to equatorial Africa after the separation of both continents. This was further supported by the phylogenetic analysis performed by Pezzini et al. [13], where they analyzed 14 *Ceiba* species in the Neotropics, indicating a Neotropical origin for the clade, with an estimated emergence during the mid-Miocene, about 21 Ma, and the divergence of *C. pentandra* at 12.7 Ma. Thus, the current knowledge indicates that *C. pentandra* migrated to west Africa and several characteristics, such as its rapid growth, tolerance to water stress, and a self-compatible mating system, could have contributed to its successful colonization of west Africa. Once established in the African moist semi-deciduous forests, *C. pentandra* was able to colonize the savannah, generating a smaller tree ecotype (about 10 m high) [5]. Some authors have proposed several subspecies in order to distinguish the American and African from the cultivated forms in Asia. However, Baker (1965) [8] (p. 6), as well as Gibbs and Semir [14] consider that the species should be considered as a single highly polymorphic species. In Africa, there is evidence that both the semi-deciduous forest and the savannah ecotypes can generate hybrids with an intermediate phenotype [8]. One of these hybrids is most likely the origin of cultivated kapok (usually referred to as *C. pentandra* var. *indica*) [5].

The introduction of *C. pentandra* into Asia is still a debated issue, with no definitive answer. However, authors, such as Baker (1965) [8] (p. 6) and Zeven [5], support the notion that the cultivated forms of *C. pentandra* come from a reduced pool of parental trees, due to the low diversity observed among the different populations present in Asia. In Figure 2, we present the migration routes towards Asia proposed by Baker (1965) [8] (p. 6) and Blench [8]. Some accounts have hypothesized that the Portuguese might have brought the species from America to Africa, and later introduced it to Asia; this was quickly dismissed due to the presence of some pictorial records depicting the species east of the Indian Ocean about 1500 years ago (Steinman 1934, in Blench [8] p. 5), before any possible incursion could be made by the Portuguese [8]. However, Steinman’s claims of the pictorial representations have also been questioned, considering that the paintings might represent some other local species bearing similarities with *C. pentandra* [8]. Other accounts propose that the species was introduced to India first and then to southwest Asia between 500 BCE and 500 CE and, supported by the pictorial representations presented by Steinman (1934) and Toxopeus (1941, in [5] pp. 271–272), has already been cultivated by the start of the 10th century [5].

## 5. Kapok Fiber Characteristics and Uses

The kapok fiber, a light fiber with a hollow tubular structure, is about 1 to 2 cm long. The fibers are comprised of microtubes with a mean external diameter of about 10 μm and a wall thickness of 0.1 μm; meanwhile cotton fibers present mean external diameter of about 16.8 μm and a wall thickness of 3.9 μm [45,46]. These characteristics, that provide less strength, were also reported for other species of *Ceiba* [47]. The cellular origin of the kapok fiber, the cells of the endocarp, facilitates fiber collection, such as cotton lint, as it is not attached to the seed. Cotton fiber originates from the epidermal cells of the seed coat [5,48]. The most common use of the fiber produced by any of the *Ceiba* species, reported in different regions of the world, is its use as fillers for pillows and cushions. However, due to their hollow structure, kapok fiber aggregates have key properties, including superhydrophobicity and porosity, ideally suited for life-saving supplies due to their maneuverability and increased buoyancy, as well as other attributes that artificial buoyancy materials lack, such as biodegradability, acid/alkali resistibility and natural abundance [45]. For textile uses, kapok fibers are short and light so kapok fiber used for fabrics or yarns must be blended with other cellulosic fibers, such as cotton or rayon, in order to improve its stability [49]. However, blending kapok with cotton or other fibers to make fabrics or yarns could reduce the amount of water and resources used during manufacturing, reducing the overall carbon footprint and environmental impact of the end product, in contrast to a similar product produced entirely of cotton or synthetic fibers. The clothing and textile industries are two of the most environmentally costly industries and face several challenges towards sustainability at every level of production [50]. Thus, diversifying prime materials and eco-friendly manufacturing alternatives will aid towards ameliorating current and future impacts. 

Kapok fibers are a potential source of cellulose and nanocellulose, comprised of up to 69% cellulose [51]. The high cellulose content has also been described in fibers of *C. speciosa* [52] and *C. aesculifolia* [47], suggesting that the fibers of the different *Ceiba* species could be an important source of cellulose and nanocellulose, polymers extensively used in biotechnological industries.

Kapok fiber is an excellent oil absorbent due to its hydrophobic nature; it has a high proportion of acetyl groups (approximately 13%). It has been suggested that this fiber could be used to recover oil spilled in water [53]. Moreover, kapok fiber as a natural material that has relatively lower cost and better biodegradability could be a better option compared to usual synthetic products [54]. 

## 6. Other Exploitable Resources from *Ceiba* Species

*C. pentandra* is also cultivated commercially for its seeds. Each kapok tree bears 1000 to 2000 pods annually that yield about 15 to 25 kg seeds. Chemical analyses of seeds has demonstrated that they are composed of 31–33% protein, 19–22% sugar and 27–28% lipids [55]. Kapok oil, which is extracted from the seeds, is used for the manufacture of the soap and as a substitute for cotton-seed oil. The use this oil as biofuel has also been proposed [44]. The most abundant fatty acids are linoleic acid, palmitic acid and oleic acid, and malvalic and sterculic acids have been also identified in *C. pentandra* and *C. speciosa* seeds [56,57].

The seeds and roots of *Ceiba aesculifolia* are commercialized as food in central Mexico, but mostly in the traditional markets of communities where these trees are found [58,59]. The seeds, bark and roots are also used traditionally to treat several illnesses, including gastritis, kidney disorders and skin infections, and to reduce blood sugar levels [58]. The bark also possesses antioxidant properties. A recent study demonstrated that the tubers of *C. aesculifolia* are edible with a good potential. These tubers contain protein (3.64%), lipids (3.18%) and carbohydrates (68.27%) [60].

## 7. High Throughput Technologies and Molecular Approaches towards Plant-Resource Management

Over the past 20 years, there has been an important increase in plant genome assemblies. However, half of the 137 land–plant orders lack a representative genome, while 6 orders are over-represented. Malvales, with 32 genomes, is one of these orders with 30 assemblies from species in the Malvaceae family. The *Gossypium* L. genus is over-represented with 22 genomes and the *G. raimondi* L. assembly was the first reported for this family in 2012 [61]. In 2018, the genome of *Bombax ceiba* L. was reported, along with the complete chloroplast and mitochondria genome sequences. The phylogenetic analysis using these genomes showed that *B. ceiba* has a close relationship with the genus *Gossypium* [62,63,64]. In 2020, the database MaGenDB was published, which included the genomic information of 13 Malvaceae species. This database could be a useful tool for comparative genomics between Malvaceae species [65]. 

Meanwhile, genetic information for *Ceiba* species is limited. Microsatellite markers were developed for *C. pentandra* in 2003, with aims to explore the mating system, genetic diversity and flow as well as other population dynamics in the Peruvian and Brazilian Amazon [25]. In 2019, the chloroplast genome of *C. speciosa* was sequenced and characterized; the phylogenetic analysis showed that *C. speciosa* was closest to *B. ceiba* [66]. In 2020, an extensive transcriptomic study from germinating seeds of *C. aesculifolia* was reported. About 54,000 transcripts were assembled, representing 12,683 complete coding transcripts with similarity to *Arabidopsis thaliana* (L.) Heynh. These transcripts represent most of the putative genes for protein synthesis that participate in the germination process as described for other species, which have been estimated in about 12,000 to 18,000 genes [36,67,68]. These germinating seed transcriptomes included information related to genes that are involved in either abiotic or biotic stress. The genes reported include LEA proteins and heat shock proteins as well as proteins involved in pathogen resistance. These data could be a valuable resource for different molecular, biochemical and cellular studies of *Ceiba* species related to drought resistance, thermotolerance and fiber development, among other processes. 

The genetic bases involved in the regulation of cotton fiber development have been extensively studied [69]. The two genes from the MYB family of transcription factors *GhMML3* and *GhMML4* have been identified to act as master regulators of cotton fiber initiation. Evolutionary analysis of this gene family revealed that these genes are grouped in two Malvaceae-specific clades and have been detected in *Theobroma cacao* L., *Durio zibethinus* Rumph. ex Murray and *B. ceiba* [70]. The germinating seed transcriptome from *C. aesculifolia* present information of MYB transcription factors. As expected, all the detected transcripts seem to be orthologous of the *A. thaliana* genes because the specific MYB genes of Malvaceae are involved in epidermal cell differentiation during fruit and seed development [71].

## 8. The Future of the Genus *Ceiba* and Perspectives

**Figure 3:**
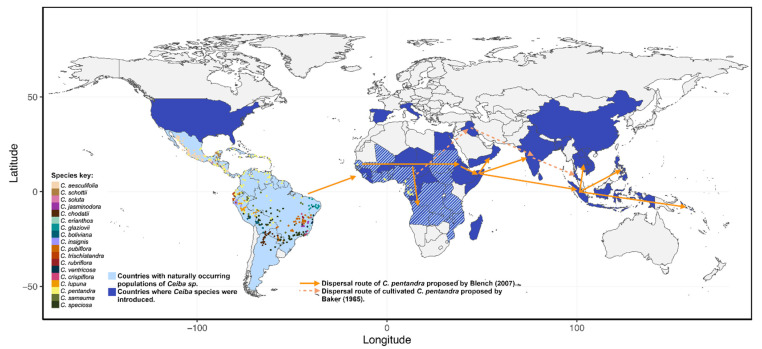
Distribution of the 18 recognized *Ceiba* species, and proposed routes of human-facilitated dispersion of *C. pentandra*. The dots represent the data of naturally-occurring specimens, adapted from Dick et al. [42] and Pezzini et al. [13]. Countries with both light and dark blue lines in Africa represent those countries where there are mixed reports of both natural populations of *C. pentandra* and assisted introductions. Dispersal routes towards Asia were adapted from Blench [8]. Data on assisted introductions was compiled from several sources, primarily [73,74].

Despite the relative cultural importance of the genus in America, there is still insufficient information on the ecology and management towards sustainable exploitation of the different resources they can offer. As seen in previous sections, most of the ecological research has been carried out in the two species widely distributed in north and central America, although the vast majority of species are located in south America (Figure 3). Moreover, in contrast to Asia, exploitation of *Ceiba* species is either in the form of being ornamental (particularly of *C. speciosa* and *C. chodatti*) or it occurs as exploitation of resources at a small scale by local communities. Therefore, there is still much work needed in order to develop sustainable exploitation in the Americas, while also implementing proper conservation and management of wild populations. In the case of *C. aesculifolia*, due to the exploitation of their reproductive structures and roots, Arellanes-Cancino et al. [72] conducted a study in the valley of Tehuacán-Cuicatlán (Mexico) in order to assess current status of wild populations and offer insight into sustainable management strategies of the species. The inhabitants of the valley have utilized and managed the species over many generations, creating a strong cultural bond with the species, although no formal cultivation occurs [58]. This instead has put some pressure over several populations found within the valley, indicating that some of them could be at risk if no proper measures are implemented [72]. Further demographic studies similar to this study are needed to monitor wild populations, as well as a closer involvement of different social actors, decision-makers and academia in order to protect both the species and the livelihood of the inhabitants of the valley.

## 9. Conclusions

Although many ecological, physiological and sustainability-oriented aspects on the biology of *Ceiba* species are still insufficient for full-scale exploitation of the genus, it is evident that there is work in process towards that end. Most of the research presented here must be replicated in understudied species, especially considering the endemic status and the risk of habitat loss that most species face. Still, the species in this genus offer an opportunity to cope with these same threats, due to their ability to withstand environmental stress and human-induced disturbances. They could also offer alternative sources of natural fibers, by designing rational and data-driven strategies for conservation and sustainable exploitation of resources. Including kapok-based fibers into the initial stages of textile manufacturing could significantly reduce the carbon footprint of the final products and reduce our dependency of cotton and oil-based fibers, two prime materials with the highest carbon footprints. However, kapok exploitation should also consider the relevance of *ad*-*hoc* strategies based on the natural diversity of *Ceiba* species and the ecological context in which they thrive, while also involving local communities, investors and governments.

## Figures and Tables

**Table 1:** Morphological characteristics, distribution and phenology of the 18 recognized species by [14,15]. Descriptions have been obtained from the aforementioned authors; information obtained from other sources is indicated in brackets. SDTF: seasonally dry tropical forest.

Species	Height	Flower Features	Trunk	Habitat	Distribution	Flowering Time	Pollinators
*C. aesculifolia* (Kunth) Britten and Baker f.	tree, 8–15 m [16]	cream, ivory	slender trunk with spines	SDTF	Mexico and Central America	March–July (September–January)	bats
*C. boliviana* Britten and Baker f.	tree, 10 m	pink with intense dark red striations	usually ventricose with spines	SDTF	Bolivia, Peru	March–April(January)	possibly bats
*C. chodatii* (Hassl.) Ravenna	tree, 12 m	ivory to pale yellow, sometimes with crimson flecks	ventricose, usually with spines	SDTF	Argentina, Bolivia and Paraguay	February–May	possibly sphingid moths
*C. crispiflora* (Kunth) Ravenna	tree, over 10 m	dark pink magenta with few striations distally, yellowish at the base	usually with spines	SDTF and humid forests	Brazil	February–March	possibly diurnal butterflies
*C. erianthos* (Cav.) K. Schum.	tree, 10 m	white with sparse carmine striations distally, becoming uniform towards the base	presents spines	SDTF	Brazil	March–July	bats
*C. glaziovii* (Kuntze) K. Schum.	tree, 10–15 m	white, sometimes with magenta striations towards the base	ventricose, with spines	SDTF	Brazil	July-September	possibly bats
*C. insignis* (Kunth) P.E. Gibbs and Semir	tree, 10 m	white to light pink with a yellowish base, occasionally with red striations	ventricose, usually with spines	SDTF	Ecuador and Peru	May–July (October)	possibly bats
*C. jasminodora* (A. St.-Hil.) K. Schum.	treelet, 1–2 m	cream, markedly reflexed	spiny branches	SDTF rocky outcrops [13]	Brazil	April–July	possibly moths
*C. lupuna* P.E. Gibbs and Semir	giant tree, up to 50 m	deep red distally, pale yellow with red speckles towards the base	usually with spines	humid forests	Ecuador, Peru and Brazil	May–June	unknown
*C. pentandra* (L.) Gaertn.	giant tree, up to 60 m; the savannah ecotype about 10 m; cultivated types 25 m [5]	white to distinctive light pink	presents spines and large buttresses (cultivated types can lack both) [5]	SDTF and humid forests [13]	pantropical, introduced in Asia and Europe	August–September	bats, possibly bees
*C. pubiflora* (A. St.-Hil.)	Tree, over 20 m	pale pink with sparse dark flecks, or pink-lilac with carmine striations	sometimes ventricose, with spines	SDTF	Paraguay, Argentina and Brazil	February–May	possibly hummingbirds
*C. rubriflora* Carv.-Sobr. and L.P. Queiroz	tree, 20 m	deep red	ventricose, with spines	SDTF, calcareous outcrops	Brazil	July–August	unknown
*C. samauma* (Mart.) K. Schum.	tree, 15 m	white but with dense golden brown trichomes	may present buttresses, spiny branches	SDTF and humid forests	Ecuador, Peru, Brazil and Bolivia	December–March (May)	unknown
*C. schottii* Britten and Baker f.	tree, 8 m	white	presents spines	SDTF, mangroves and flood zones [17]	Mexico and Central America	June–October [17]	diurnal butterflies
*C. soluta* (Donn. Sm.) Ravenna	Not reported	white	presents spines	SDTF	Guatemala	February	unknown
*C. speciosa* (A. St.-Hil.) Ravenna	tree, 10–20 m	dark pink magenta distally, base white to yellow, usually with dark striations	ventricose, usually with spines	SDTF and humid forests	Argentina, Bolivia and Brazil	January–May	possibly diurnal butterflies
*C. trischistandra* (A. Gray) Bakh.	tree, 15–30 m	white, externally tinted green, somewhat reflexed	presents spines	SDTF	Ecuador	April-July	unknown
*C. ventricosa* (Nees and Mart.) Ravenna	tree, 10 m or more	white to cream with dark redish flecks towards the base	ventricose, usually with spines	SDTF	Brazil	February-April	possibly bats

## Data Availability

Not applicable.

## References

[1] Townsend T., Sette J., Fangueiro R., Rana S. (2016). Natural Fibres and the World Economy. Natural Fibres: Advances in Science and Technology towards Industrial Applications.

[2] Chen H.L., Burns L.D. (2006). Environmental analysis of textile products. CTRJ.

[3] Gedik G., Avinc O., Muthu S.S., Gardetti M.A. (2020). Hemp fiber as a sustainable raw material source for textile industry: Can we use its potential for more eco-friendly production?. Sustainability in the Textile and Apparel Industries.

[4] Carvalho-Sobrinho J.G., Alverson W.S., Alcantara S., Queiroz L.P., Mota A.C., Baum D.A. (2016). Revisiting the phylogeny of Bombacoideae (Malvaceae): Novel relationships, morphologically cohesive clades, and a new tribal classification based on multilocus phylogenetic analyses. Mol. Phylogenet. Evol..

[5] Zeven A.C., Ferwerda F.P., Wit F. (1969). Kapok tree, *Ceiba pentandra* Gaertn. Outlines of Perennial Crop Breeding in the Tropics.

[6] Zheng Y., Wang J., Wang A. (2021). Recent advances in the potential applications of hollow kapok fiber-based functional materials. Cellulose.

[7] Food and Agriculture Organization of the United Nations FAOSTAT Statistical Database. https://www.fao.org/faostat/en/#home.

[8] Blench R.M., Cappers R.T.J. (2007). The intertwined history of the silk-cotton and baobab. Fields of Change: Progress in African Archaeobotany.

[9] Barrera-Vázquez A. (1975). La ceiba-cocodrilo. Anales INAH.

[10] López-Austin A. (2019). Las cuatro columnas. Arqueol. Mex..

[11] Comisión Nacional de Áreas Naturales Protegidas (2021). De Mitos y Leyendas, la Ceiba. https://www.gob.mx/conanp.

[12] Hurtado V. (2021). Datos Interesantes Sobre Algunos de los Símbolos Patrios de Guatemala. https://flaar-mesoamerica.org.

[13] Pezzini F.F., Dexter K.G., de Carvalho-Sobrinho J.G., Kidner C.A., Nicholls J.A., De Queiroz L.P., Pennington R.T. (2021). Phylogeny and biogeography of *Ceiba* Mill. (Malvaceae, Bombacoideae). Front. Biogeogr..

[14] Gibbs P., Semir J. (2003). A taxonomic revision of the genus *Ceiba* Mill. (Bombacaceae). Anales Jard. Bot. Madrid.

[15] De Carvalho-Sobrinho J.G., de Queiroz L.P. (2008). *Ceiba rubriflora* (Malvaceae: Bombacoideae), a new species from Bahia, Brazil. Kew Bull..

[16] Niembro-Rocas A., Vázquez-Torres M., Sánchez-Sánchez O. (2010). Árboles de Veracruz: 100 Especies Para la Reforestación Estratégica.

[17] Duno de Stefano R., Carnevali Fernández-Concha G., Ramírez Morillo I.M., Tapia Muñoz J.L., Can Itzá L.L., Hernández-Aguilar S., Embray T. (2010). Flora de la Península de Yucatán. https://www.cicy.mx/sitios/flora%20digital/ficha_virtual.php?especie=1746.

[18] Bullock S.H., Mooney H.A., Medina E. (1995). Seasonally Dry Tropical Forests.

[19] Dirzo R., Young H.S., Mooney H.A., Ceballos G. (2011). Seasonally Dry Tropical Forests: Ecology and Conservation.

[20] Linares-Palomino R., Oliveira-Filho A.T., Pennington R.T., Dirzo R., Young H.S., Mooney H.A., Ceballos G. (2011). Neotropical Seasonally Dry Forests: Diversity, Endemism, and Biogeography of Woody Plants. Seasonally Dry Tropical Forests.

[21] Lopezaraiza-Mikel M., Quesada M., Álvarez-Añorve M., Ávila-Cabadilla L., Martén-Rodríguez S., Calvo-Alvaado J., do Espírito-Santo M.M., Fernandes G.W., Sánchez-Azofeifa A., Aguilar-Aguilar M.J., Sanchez-Azofeifa A., Powers J.S., Fernandes G.W., Quesada M. (2013). Phenological patterns of tropical dry forests along latitudinal and successional gradients in the neotropics. Tropical Dry Forests in the Americas.

[22] Maas M., Burgos A., Dirzo R., Young H.S., Mooney H.A., Ceballos G. (2011). Water dynamics and the ecosystem level in seasonally dry tropical forests Seasonally Dry Tropical Forests: Ecology and Conservation. Seasonally Dry Tropical Forests.

[23] Herrerías-Diego Y., Quesada M., Stoner K.E., Lobo J.A. (2006). Effects of forest fragmentation on phenological patterns and reproductive success of the tropical dry forest tree *Ceiba aesculifolia*. Conserv. Biol..

[24] Valle-Díaz O., Blanco-García A., Bonfil C., Paz H., Lindig-Cisneros R. (2009). Altitudinal range shift detected through seedling survival of *Ceiba aesculifolia* in an area under the influence of an urban heat island. Forest Ecol. Manag..

[25] Brondani R.P.V., Gaiotto F.A., Missiaggia A.A., Kirst M., Gribel R., Grattapaglia D. (2003). Microsatellite markers for *Ceiba pentandra* (Bombacaceae), an endangered tree species of the Amazon Forest. Mol. Ecol. Notes.

[26] IUCN 2022 The International Union for Conservation of Nature and Natural Resources Red List of Threatened Species. https://www.iucnredlist.org/.

[27] Cusack D.F., Karpman J., Ashdown D., Cao Q., Ciochina M., Halterman S., Lydon S., Neupane A. (2016). Global change effects on humid tropical forests: Evidence for biogeochemical and biodiversity shifts at an ecosystem scale. Rev. Geophys..

[28] Velázquez-Rosas N., Ruiz-Guerra B., Sánchez-Coronado M.E., Gamboa de Buen A., Orozco-Segovia A. (2017). Morphological variation in fruits and seeds of *Ceiba aesculifolia* and its relationship with germination and seedling biomass. Bot. Sci..

[29] Martínez-González I., Sánchez-Velázquez L.R., Ruiz-Guerra B., del Rosario Pineda-López M., Velázquez-Rosas N. (2021). The role of seed size in the emergence and survival of seedlings in contrasting environments: The case of *Ceiba aesculifolia*. New For..

[30] Vargas-Rodriguez Y.L., Vázquez-García J.A., Williamson G.B. (2005). Environmental correlates of tree and seedling–sapling distributions in a Mexican tropical dry forest. Plant Ecol..

[31] Pineda-García F., Paz H., Meinzer F.C. (2013). Drought resistance in early and late secondary successional species from a tropical dry forest: The interplay between xylem resistance to embolism, sapwood water storage and leaf shedding. Plant Cell Environ..

[32] Mendoza P.A.M., Moya E.G., Rivera J.R.A., Xolocotzi E.H. (1995). Regeneración natural de especies arbóreas en una selva mediana subperennifolia perturbada por extracción forestal. Acta Bot. Mex..

[33] Encino-Ruiz L., Lindig-Cisneros R., Gómez-Romero M., Blanco-García A. (2013). Desempeño de tres especies arbóreas del bosque tropical caducifolio en un ensayo de restauración ecológica. Bot. Sci..

[34] Martínez-González I., Velázquez-Rosas N., del Rosario Pineda-López M., Ruiz-Guerra B., Sánchez-Velásquez L.R. (2021). The role of seed size in the performance of *Ceiba aesculifolia* seedlings subjected to foliar damage. Acta Oecol..

[35] Olvera-Mendoza E.I., Lara-Cabrera S.I., Sáenz-Romero C., Lindig-Cisneros R. (2016). AFLP polymorphism in restored provenances of *Ceiba aesculifolia* within an urban heat island. Phyton Inter. J. Exp. Bot..

[36] Gómez-Maqueo X., Soriano D., Velázquez-Rosas N., Alvarado-López S., Jiménez-Durán K., del Mar Garciadiego M., Gamboa-deBuen A. (2020). The seed water content as a time-independent physiological trait during germination in wild tree species such as *Ceiba aesculifolia*. Sci. Rep..

[37] Hending D., Randrianarison H., Holderied M., McCabe G., Cotton S. (2021). The kapok tree (*Ceiba pentandra* (L.) Gaertn, Malvaceae) as a food source for native vertebrate species during times of resource scarcity and its potential for reforestation in Madagascar. Austral Ecol..

[38] Immanuel R.R., Ganapathy M. (2007). Growth and physiological attributes of *Ceiba pentandra* (L.) Gaertn. seeds and seedlings under salt stress. J. Agric. Biol. Sci..

[39] Immanuel R.R., Ganapathy M. (2019). Agro-techniques for afforestation of degraded coastal agricultural lands with silk cotton (*Ceiba pentendra* (L.) Gaertn.). J. Pharmacog. Phytochem..

[40] (2021). The University of Arizona, Campus Arboretum. https://apps.cals.arizona.edu/.

[41] Guillot D., Laguna E., Puche C., Meer P. (2016). *Ceiba speciosa* ‘Valencian Beauty’. Bouteloua.

[42] Dick C.W., Bermingham E., Lemes M.R., Gribel R. (2007). Extreme long-distance dispersal of the lowland tropical rainforest tree *Ceiba pentandra* L.(Malvaceae) in Africa and the Neotropics. Mol. Ecol..

[43] Rahmah A.U., Abdullah M.A. (2011). Evaluation of Malaysian *Ceiba pentandra* (L.) Gaertn. for oily water filtration using factorial design. Desalination.

[44] Silitonga A.S., Ong H.C., Mahlia T.M.I., Masjuki H.H., Chong W.T. (2013). Characterization and production of *Ceiba pentandra* biodiesel and its blends. Fuel.

[45] Zhang X., Fu W., Duan C., Xiao H., Shi M., Zhao N., Xu J. (2013). Superhydrophobicity determines the buoyancy performance of kapok fiber aggregates. Appl. Surf. Sci..

[46] Graham B.P., Haigler C.H. (2021). Microtubules exert early, partial, and variable control of cotton fiber diameter. Planta.

[47] Carranza-Nuñez U., Ramiro Vasquez-Garcia S., Flores-Ramirez N., Ahmed Abdel-Gawwad H., Luis Rico J., Arizbe Santiago A., Vargas J., Cruz-de-León J. (2021). Physicochemical characterization of natural fibers obtained from seed pods of *Ceiba aesculifolia*. BioResources.

[48] Anderson D.B., Kerr T. (1938). Growth and structure of cotton fiber. Ind. Eng. Chem..

[49] Yan J., Xu G., Wang F. (2013). A study on the quality of kapok blended yarns through different processing methods. J. Tex. Ins..

[50] Patwary S. (2020). Clothing and Textile Sustainability: Current State of Environmental Challenges and the Ways Forward. Text. Leather Rev..

[51] Sartika D., Syamsu K., Warsiki E., Fahma F., Arnata I.W. (2021). Nanocrystalline cellulose from kapok fiber (*Ceiba pentandra*) and its reinforcement effect on alginate hydrogel bead. Starch-Stärke.

[52] Leal M.R., Flores-Sahagun T.H.S., Franco T.S., Muniz G.I. (2021). *Ceiba speciosa* St. Hill fruit fiber as a potential source for nanocellulose production and reinforcement of polyvinyl acetate composites. Polym. Compos..

[53] Hori K., Flavier M.E., Kuga S., Lam T.B.T., Iiyama K. (2000). Excellent oil absorbent kapok *Ceiba pentandra* (L.) Gaertn. fiber: Fiber structure, chemical characteristics, and application. J. Wood Sci..

[54] Thilagavathi G., Karan C.P., Thenmozhi R. (2018). Development and investigations of kapok fiber based needle punched nonwoven as eco-friendly oil sorbent. J. Nat. Fibers.

[55] Nkouam G.B., Adjoh G.A., Tchankou Leudeu C.B., Kouebou C., Tchiegang C., Kapseu C. (2017). Physico-chemical properties of fruits, seed and oil of kapok (*Ceiba pentandra* Gaertn.) tree of different provenances from the northern part of Cameroon. Int. J. Agric. Innov. Res..

[56] Berry S.K. (1979). The characteristics of the kapok (*Ceiba pentadra* Gaertn.) seed oil. Pertanika J. Trop. Agric. Sci..

[57] Rosselli S., Tundis R., Bruno M., Leporini M., Falco T., Gagliano Candela R., Badalamenti N., Loizzo M.R. (2020). *Ceiba speciosa* (A. St.-Hil.) seeds oil: Fatty acids profiling by GC-MS and NMR and bioactivity. Molecules.

[58] Avendaño A., Casas A., Dávila P., Lira R. (2006). Use forms, management and commercialization of “pochote” *Ceiba aesculifolia* (HB & K.) Britten & Baker f. subsp. parvifolia (Rose) PE Gibbs & Semir (Bombacaceae) in the Tehuacán Valley, Central Mexico. J. Arid Environ..

[59] Avendaño A., Casas A., Dávila P., Lira R. (2009). In situ management and patterns of morphological variation of *Ceiba aesculifolia* subsp. parvifolia (Bombacaceae) in the Tehuacán-Cuicatlán Valley. Econ. Bot..

[60] Suastegui-Baylón L., Salazar R., Maldonado-Astudillo Y.I., Ramírez-Sucre M.O., Arámbula-Villa G., Flores-Casamayor V., Jiménez-Hernández J. (2021). Physical, chemical and rheological characterization of tuber and starch from *Ceiba aesculifolia* subsp. parvifolia. Molecule.

[61] Marks R.A., Hotaling S., Frandsen P.B., VanBuren R. (2021). Representation and participation across 20 years of plant genome sequencing. Nat. Plants.

[62] Gao Y., Wang H., Liu C., Chu H., Dai D., Song S., Yu L., Han L., Fu Y., Tian B. (2018). De novo genome assembly of the red silk cotton tree (*Bombax ceiba*). GigaScience.

[63] Gao Y., Wang H., Liu C., Chu H., Yan Y., Tang L. (2018). Complete chloroplast genome sequence of the red silk cotton tree (*Bombax ceiba*). Mitochondrial DNA Part B.

[64] Gao Y., Wang H., Liu C., Chu H., Yan Y., Tang L. (2018). The complete mitochondrial genome of *Bombax ceiba*. Mitochondrial DNA Part B.

[65] Wang D., Fan W., Guo X., Wu K., Zhou S., Chen Z., Li D., Wang K., Zhu Y., Zhou Y. (2020). MaGenDB: A functional genomics hub for Malvaceae plants. Nucleic Acids Res..

[66] Huang S., Zhu Q., Huang G., Han B., Zhou Q., Dai J. (2019). The chloroplast genome of silk floss tree (*Ceiba speciosa*). Mitochondrial DNA Part B.

[67] Nakabayashi K., Okamoto M., Koshiba T., Kamiya Y., Nambara E. (2005). Genome-wide profiling of stored mRNA in *Arabidopsis thaliana* seed germination: Epigenetic and genetic regulation of transcription in seed. Plant J..

[68] Howell K.A., Narsai R., Carroll A., Ivanova A., Lohse M., Usadel B., Miller A.H., Whelan J. (2009). Mapping metabolic and transcript temporal switches during germination in rice highlights specific transcription factors and the role of RNA instability in the germination process. Plant Physiol..

[69] Huang G., Huang J.Q., Chen X.Y., Zhu Y.X. (2021). Recent advances and future perspectives in cotton research. Annu. Rev. Plant Biol..

[70] Wu H., Tian Y., Wan Q., Fang L., Guan X., Chen J., Hu Y., Ye W., Zhang H., Guo W. (2018). Genetics and evolution of MIXTA genes regulating cotton lint fiber development. New Phytol..

[71] Gómez-Maqueo X.M., Gamboa-deBuen A. (2022). Personal communication.

[72] Arellanes-Cancino Y., Romero-Sosa M.Á., Vega E., Maza-Villalobos S., Casas-Fernández A. (2018). Ecological bases for sustainable management of Pochote (*Ceiba aesculifolia* subsp. parvifolia) through demographic analysis. Econ. Bot..

[73] Royal Botanic Gardens Kew Plants of the World Online Database. https://powo.science.kew.org/.

[74] Tropicos.org Missouri Botanical Garden. https://tropicos.org.

